# Biodiversity, Phylogeny, and Evolutionary Dynamics of Gall Midges on Japanese Beech Trees

**DOI:** 10.1002/ece3.71621

**Published:** 2025-06-27

**Authors:** Shinnosuke Mori, Yugo Dhakhwa, Makoto Tokuda, Yoko Saikawa

**Affiliations:** ^1^ Faculty of Science and Technology Keio University Kanagawa Japan; ^2^ Faculty of Agriculture Saga University Saga Japan; ^3^ The United Graduate School of Agricultural Sciences Kagoshima University Kagoshima Japan

**Keywords:** adaptive radiation, biogeography, co‐evolution, gall midge, molecular phylogeny

## Abstract

The Japanese Archipelago harbors unique beech flora (*Fagus* L.; Fagaceae), with a parapatric distribution of two endemic species, *F. crenata* and *F. japonica* , upon which a diverse array of 34 types of leaf galls induced by gall midges (Diptera: Cecidomyiidae) has been documented. The inducers of most of these galls remain undescribed, and their phylogenetic relationships with known taxa are still poorly understood. In this study, we collected 29 types of leaf galls from the two Japanese *Fagus* species, including 6 previously unreported types, and sequenced the *cytochrome c oxidase subunit I* mitochondrial gene of the inducers to infer maximum‐likelihood and Bayesian time‐calibrated phylogenies. Our phylogenetic analyses revealed that the *Fagus*‐feeding guild forms a monophyletic clade within the tribe Dasineurini, with *F. crenata‐*feeding taxa occupying a basal position within the lineage. These taxa are closely related to the genera *Hartigiola* and *Mikiola* and have likely undergone adaptive radiation on the leaves of *F. crenata* and *F. japonica* in the ecologically segregated Japanese Archipelago since the Miocene period, accompanied by multiple host shifts between the two *Fagus* species and location shifts within their leaves.

## Graphical abstract

Leaf galls of 29 types were collected from Japanese endemic beech trees (*Fagus* L.). Most of the gall inducers formed a monophyletic clade within the tribe Dasineurini. The *Fagus*‐feeding gall midges underwent adaptive radiation since the Miocene, accompanied by multiple host shifts between *F. crenata* and *F*. *japonica*.

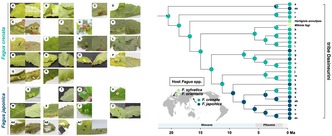

## 1 Introduction

Insects are one of the most diverse groups in terrestrial ecosystems, and almost half of all extant species are phytophagous (Price et al. 2011). Understanding the processes of speciation and adaptive radiation of phytophagous insects is thus important in order to elucidate the mechanisms of biodiversity generation.

Insect galls are malformed plant structures induced by insects such as gall midges and gall wasps, which use the galls as their own microhabitats. Galls can form on leaves, stems, floral buds, flowers, fruits, or roots and often exhibit distinctive form and color. Gall midges (Diptera: Cecidomyiidae) represent the most speciose group of galling arthropods in the world, comprising 6651 known species and thousands of undescribed species (Gagné and Jaschhof 2021). The currently accepted systematic division of Cecidomyiidae into subfamilies and tribes, as outlined in ‘A Catalog of the Cecidomyiidae (Diptera) of the World’, is based on comparative studies of morphological characteristics (Gagné and Jaschhof 2021) and corroborated by recent phylogenetic studies (Sikora et al. 2019; Dorchin et al. 2019). The Cecidomyiidae family includes six subfamilies, as follows: five basal subfamilies (Catotrichinae, Lestremiinae, Micromyinae, Winnertziinae, and Porricondylinae) that feed on fungi, and the Cecidomyiinae, the largest and youngest subfamily, which includes fungivorous, herbivorous, and predatory species. Approximately 75% of species in the Cecidomyiinae are herbivorous and diversified significantly during the Tertiary period, coevolving with angiosperms (Gagné and Jaschhof 2021). Most herbivorous gall midges are host‐specific and develop on one or a few closely related host plants, and many genera and even tribes have evolved and diversified on plants of particular families (Gagné and Jaschhof 2021).

Beech (*Fagus* L.; Fagaceae), a representative genus of tree species of deciduous forests in temperate areas of the Northern Hemisphere (Peters 1997), serves as a host for cecidomyiids of the subfamily Cecidomyiinae. The genus *Fagus* includes two distinct subgenera, *Fagus* and *Engleriana*, which include 7–10 and 3 species, respectively (Denk et al. 2005), although the number and rank of several taxa remain controversial (e.g., Gömöry et al. 2018). Regarding trees of the subgen. *Fagus*, gall midges have been found to infest Siebold's beech ( *F. crenata* Blume) in Japan, Oriental beech ( *F. orientalis* Lipsky) in areas around the Caspian Sea and Black Sea, and European beech ( *F. sylvatica* L.) in Europe (Yukawa et al. 2021). The *Fagus*‐feeding gallers comprise six genera across three tribes, as follows: *Contarinia* (Cecidomyiini), *Hartigiola*, *Janetiella*, *Macrolabis*, *Mikiola* (Dasineurini), and *Phegomyia* (Trotteriini) (Yukawa et al. 2021). By contrast, field surveys in North America reported the absence of cecidomyiid galls on American beech ( *F. grandifolia* Ehrhart) and Mexican beech ( *F. mexicana* Martinez) (Sato and Yukawa 2001). In subgen. *Engleriana*, Japanese beech ( *F. japonica* Maximowicz) is the only species infested by cecidomyiids, with *Hartigiola annulipes* (Hartig) as a described species.

The Japanese Archipelago is home to unique beech flora due to the parapatric distribution of two endemic species, each of which belongs to a different subgenus: *F. crenata* (subgen. *Fagus*) and *F. japonica* (subgen. *Engleriana*). At least 26 types of leaf galls have been documented on *F. crenata* , with 8 types documented on *F. japonica* (summarized in Sato et al. 2010). As gall morphology is essentially species‐specific (Yukawa and Masuda 1996), formation of the respective types of galls is thought to be induced by different cecidomyiid species. *Fagus crenata* harbors the greatest abundance of gall‐inducing cecidomyiids in the Japanese Archipelago (Yukawa and Masuda 1996), and elucidating their speciation processes will be necessary in order to understand the diversification of gall inducers in relation to specific host plants. Among cecidomyiids associated with *F. crenata* and *F. japonica* , six species have been described and identified to date: *Mikiola bicornis* Sato and Yukawa, *Mikiola glandaria* Sato and Yukawa, *Hartigiola faggalli* (Monzen), *Hartigiola annulipes* (Hartig), *Janetiella infrafoli* Monzen, and *Phegomyia tokunagai* Sasakawa and Koyama (Table 1; Sato et al. 2010), all of which belong to the supertribe Lasiopteridi of subfamily Cecidomyiinae (Gagné and Jaschhof 2021). Although the nucleotide sequences of *cytochrome c oxidase subunit I* (*COI*), a ‘DNA barcode’, are publicly available for *Hartigiola faggalli* and *Hartigiola annulipes*, sequence data for other gall‐inducing cecidomyiid species are lacking; thus, this lack of molecular data hampers reconstruction of the evolutionary relationships among gall‐inducing cecidomyiids, hindering efforts to elucidate lineage diversification and potential adaptive radiations. This study therefore addressed this knowledge gap by collecting *COI* sequences from *Fagus*‐feeding cecidomyiids that induce 29 types of galls, including previously unreported types, in order to infer the molecular phylogeny within the Cecidomyiinae. The study also investigated the taxonomy, evolutionary context, and larval feeding modes of these cecidomyiids and explored the relationship between the morphological diversity of galls and genetic variations in gall inducers within a phylogenetic framework.

**TABLE 1:** Types of galls on the leaves of Japanese *Fagus* trees ( *F. crenata* and *F. japonica* ), gall inducers, gall sites, and month of collection.

Host plant	No.	Gall ID	Description of gall	Japanese gall name^b^	Inducer ID	Gall midge^b^	Site	Month
*F. crenata*	1	**A**	Pocket gall along the side vein	Buna‐hamyaku‐kobufushi	**a**	Undescribed	N	May, Sep
	2	**B**	Conical gall	Buna‐hasuji‐togaritamafushi	**b**	*Mikiola bicornis* Sato and Yukawa^b^	G, N	May, Sep
	3	—		Buna‐hasuji‐dongurifushi	—	*Mikiola glandaria* Sato and Yukawa^b^	—	—
	4	**C**	Globular gall on the leaf edge	Buna‐haberi‐tamafushi	**c**	Undescribed	G, N	May, Sep
	5	**D**	Horn‐shaped gall on the rolled leaf edge	Buna‐haberi‐hosofushi	**d**	Undescribed	A, G	Apr, May
	6	**E**	Bivalve‐shaped gall (abaxial side)	Buna‐haura‐kaigarafushi	**e**	*Hartigiola faggalli* (Monzen)^c^, ^d^	M	Oct
	7	—		Buna‐haura‐kefushi	—	Undescribed	—	—
	8	—		Buna‐haura‐kobufushi	—	Undescribed	—	—
	9	**F**	Hairy gall at the intersection of veins	Buna‐haura‐kometsubufushi	**f**	*Janetiella infrafoli* Monzen^b^	M, N	Sep, Oct
	10	—		Buna‐haura‐hishigatafushi	—	Undescribed	—	—
	11	**G**	Globular gall with red hair	Buna‐ha‐akagetamafushi	**g**	Undescribed	A, G, K, N	Apr, May
	12	—		Buna‐ha‐ootsunofushi	—	Undescribed	—	—
	13	**H**	Bivalve‐shaped gall (adaxial side)	Buna‐ha‐kaigarafushi	**h**	*Hartigiola faggalli* (Monzen)^c^, ^d^	M, N	Sep, Oct
	14	**I**	Fang‐shaped gall	Buna‐ha‐kibatsunofushi	**i**	Undescribed	M	Oct
	15	—		Buna‐ha‐ketamafushi	—	Undescribed	—	—
	16	**J**	Small horn‐shaped gall	Buna‐ha‐kotsunofushi	**j**	Undescribed	A, K	May
	17	**K**	Globular gall with smooth surface	Buna‐ha‐tamafushi	**k**	Undescribed	A, K, N	May, Sep
	18	—		Buna‐ha‐tsunofushi	—	Undescribed	—	—
	19	—		Buna‐ha‐togetsunofushi	—	Undescribed	—	—
	20	**L**	Horn‐shaped gall	Buna‐ha‐nagatsunofushi	**l**	Undescribed	N	Sep
	21	**M**	Nodular gall	Buna‐ha‐fukurefushi	**m**	Undescribed	K, G, N	May, Sep
	22	**N**	Thick horn‐shaped gall	Buna‐ha‐futotsunofushi	**n**	Undescribed	A, G, K	May
	23	—		Buna‐ha‐hosotogaritamafushi	—	*Phegomyia tokunagai* Sasakawa and Koyama^b^	—	—
	24	**O**	Globular gall with a constricted base	Buna‐ha‐magetamafushi	**o**	Undescribed	A, K, G, N	Apr, May
	25	—		Buna‐ha‐marutamafushi	—	Undescribed	—	—
	26	**P**	Horn‐shaped gall with a rounded tip	Buna‐ha‐marutsunofushi	**p**	Undescribed	M, N	Sep, Oct
	27	**Q**	Horn‐shaped gall on the leaf edge^a^	Buna‐haberi‐tsunofushi	**q**	Undescribed	A	May
	28	**R**	Nodular gall on the leaf edge^a^	Buna‐haberi‐kobufushi	**r**	Undescribed	A, K	May
*F. japonica*	29	**S**	Globular gall on the leaf edge	Inubuna‐haberi‐tamafushi	**s**	Undescribed	G, M	Jun, Sep
	30	—		Inubuna‐haberi‐hosofushi	—	Undescribed	—	—
	31	**T**	Pocket gall along the side vein	Inubuna‐hamyaku‐kobufushi	**t**	Undescribed	G	Apr, May, Jun
	32	**U**	Globular gall with red hair	Inubuna‐ha‐akagetamafushi	**u**	Undescribed	G	May
	33	**V**	Cylindrical gall with white hair	Inubuna‐ha‐ketsunofushi	**v**	*Hartigiola annulipes* (Hartig)^c^	G	May, Jun, Aug, Sep
	34	**W**	Horn‐shaped gall with a rounded tip	Inubuna‐ha‐tsunofushi	**w**	Undescribed	G	May, Jun
	35	**X**	Button‐shaped gall	Inubuna‐ha‐botanfushi	**x**	Undescribed	G	Aug
	36	**Y**	Globular gall with smooth surface	Inubuna‐ha‐marutamafushi	**y**	Undescribed	G, M	May, Jun, Sep
	37	**Z**	Fang‐shaped gall^a^	Inubuna‐ha‐kibatsunofushi	**z**	Undescribed	G	Apr, May
	38	**AA**	Elongated pocket gall along the side vein^a^	Inubuna‐hamyaku‐nagakobufushi	**aa**	Undescribed	G	Apr
	39	**AB**	Onion‐shaped gall on the leaf edge^a^	Inubuna‐haberi‐giboshifushi	**ab**	Undescribed	M	Sep
	40	**AC**	Ovoid gall with a hairy surface^a^	Inubuna‐ha‐kekotamafushi	**ac**	Undescribed	M	Sep

*Note:* Collection sites are abbreviated as follows: A, Mt. Amagi; G, Mt. Gozen; K, Kanyudo; M, Mt. Mito; N, Mt. Nabewari.

^a^ Types newly reported in this study.

^b^ No sequence deposited in any public database.

^c^ *COI* sequence available in GenBank (Note: the *COI* sequence identity between *H. annulipes* [MN191300.1] collected from a gall on *F. sylvatica* in Germany and **v** was 90.1% [547/607 bp]).

^d^ The gall midges inducing these two types (morphotypes) are currently regarded as the same species (Sato and Yukawa 2004).

## 2 Materials and Methods

### 2.1 Gall Midge Collection

**FIGURE 1:**
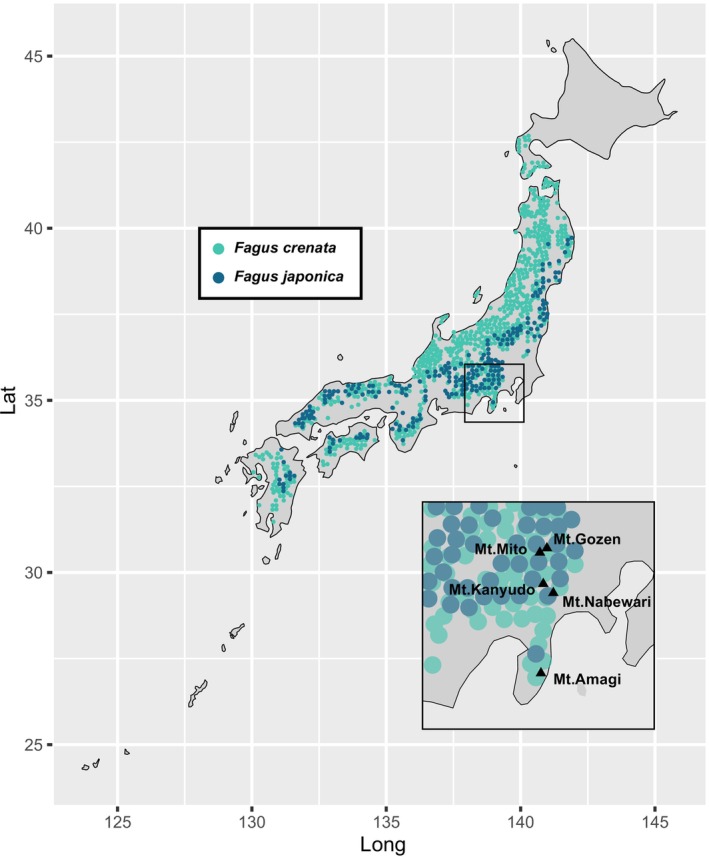
Geographical distribution of *F. crenata* and *F. japonica* and collection sites. Reproduced from Phytosociological Relevé Database (Tanaka and Matsui 2007). The map was generated using the R (R Core Team 2024) ‘maps’ package version 3.3.0 [Original S code by Richard A. Becker, Allan R. Wilks. R version by Ray Brownrigg. Enhancements by Thomas P Minka and Alex Deckmyn (2018) maps: Draw Geographical Maps. https://CRAN.R‐project.org/package=maps].

**FIGURE 2:**
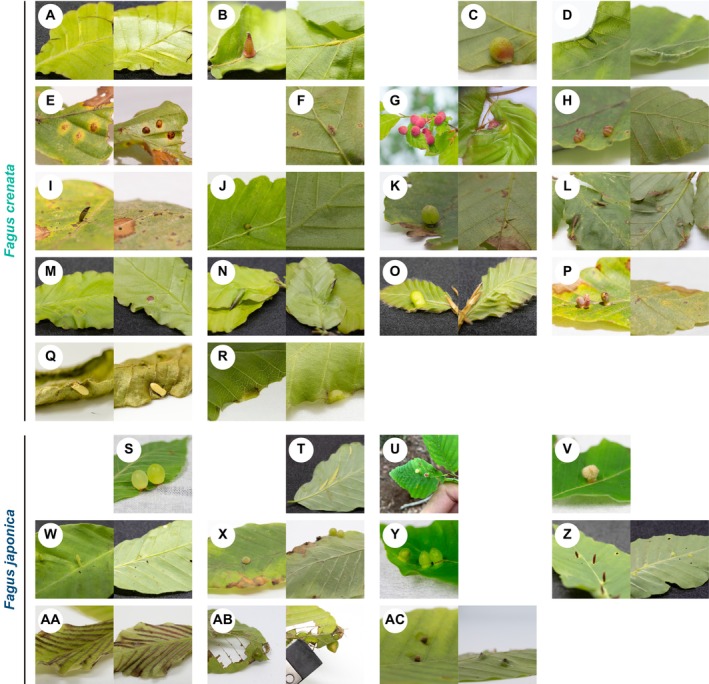
Types of galls found on the leaves of *F. crenata* and *F. japonica* in this study. Leaf galls (**A–R**) on *F. crenata* and (**S–AC**) on *F. japonica* (see Table 1 for detailed descriptions of each gall). (**U**) The red gall on the right is **U**, and the white one on the left is **V**. View from the adaxial side (left) and abaxial side (right) in each panel.

A total of 29 types of galls (gall ID, **A**–**AC**) were collected from leaves of *F. crenata* and *F. japonica* specimens across five mountainous areas of the Kanto and Chubu regions of Japan (Figures 1 and 2; Table 1): Mt. Amagi (elevation, 1406 m; 34°51′30.6″N, 139°0′27.6″E) in Shizuoka, Mt. Gozen (elevation, 1405 m; 35°46′18.4″N, 139°4′51.0″E) in Tokyo, Mt. Kanyudo (elevation, 1418 m; 35°30′40.1″N, 139°0′49.0″E) and Mt. Nabewari (elevation, 1272 m; 35°26′39.5″N, 139°8′31.6″E) in Kanagawa, and Mt. Mito (elevation, 1531 m; 35°43′53.0″N, 138°58′37.4″ E) in Yamanashi. Galls were collected during the periods April–June and August–October of 2022 to 2024. On Mt. Gozen and Mt. Mito, *F. crenata* and *F. japonica* grow sympatrically at a local scale, whereas no *F. japonica* were observed on Mt. Amagi, Mt. Kanyudo, or Mt. Nabewari.

All types of galls found on *F. crenata* and *F. japonica* were classified as shown in Table 1 according to Yukawa (1991), Yukawa and Masuda (1996), and Sato and Yukawa (2004). Of the previously documented gall types listed in Table 1, those without IDs were not found during our investigation. Six types of galls (**Q**, **R**, and **Z**–**AC**) exhibited previously unreported morphologies, for which Japanese names were assigned based on the characteristics (Table 1). Gall specimens were photographed either at the collection site or in the laboratory before collection of gall midges. To collect midges, galls were gently excised using a scalpel blade, and larvae or pupae (inducer ID, **a**–**ac**) within the galls were collected under a stereomicroscope. Gall midge specimens were preserved in 99.5% ethanol at 4°C until use. When multiple galls of the same type were available, 2–5 cecidomyiid individuals were collected per type and distinguished by adding hyphenated numbers (e.g., b‐1, b‐2, and b‐3) to the corresponding inducer ID (**a**–**ac**). For seven types (**A**, **F**, **L**, **Q**, **T**, **V**, and **X**), however, only a single specimen could be collected due to the scarcity in the field.

### 2.2 DNA Extraction, Amplification, Sequencing, and Editing

Larval and pupal specimens were homogenized using a BioMasher II (Takara Bio, Tokyo, Japan), and gDNA was extracted from the homogenized samples using NucleoSpin DNA Insect (Macherey‐Nagel, Düren, Germany), following the manufacturer's instructions.

The extracted DNA was subjected to PCR to amplify approx. 700 bp within the mitochondrial *COI* gene using the primer set LCO1490 (5′‐GGT CAA CAA ATC ATA AAG ATA TTG G‐3′) and HCO2198 (5′‐TAA ACT TCA GGG TGA CCA AAA AAT CA‐3′) (Folmer et al. 1994). The primers were synthesized by Eurofins Genomics (Tokyo, Japan). Amplification was performed using KOD FX DNA polymerase (Toyobo, Osaka, Japan) and a MiniAmp Thermal Cycler (Thermo Fischer Scientific, MA, USA) under the following conditions: initial denaturation at 94°C for 2 min, followed by 40 cycles of denaturation at 98°C for 10 s, annealing at 40°C for 30 s, and extension at 68°C for 15 s. For samples where amplification was insufficient, modified conditions were applied using an annealing temperature of 50°C or 60°C and/or an extension time of 30 s.

PCR amplification products were cleaned up enzymatically using ExoSAP‐IT PCR Product Cleanup (Thermo Fischer Scientific) or purified using either NucleoSpin Gel and PCR Clean‐up XS (Macherey‐Nagel) or the E‐Gel Power Snap Electrophoresis System with CloneWell II 0.8% agarose gels (Thermo Fischer Scientific). The amplicons were then bidirectionally Sanger‐sequenced by Eurofins Genomics using the same primers described above. Forward and reverse sequences were manually edited with reference to the chromatograms and assembled into consensus sequences using 4Peaks ver. 1.8 (https://nucleobytes.com/4peaks/index.html). Sequence ends exhibiting ambiguity were trimmed. The nucleotide sequence data generated in this study were deposited in the NCBI GenBank nucleotide sequence database under the accession numbers listed in Table S1 (accession, PQ838091–PQ838157).

### 2.3 Phylogenetic Analysis

A phylogenetic analysis was conducted on the collected cecidomyiids within the subfamily Cecidomyiinae, which includes previously reported species that infest *Fagus* species (*Contarinia*, *Janetiella*, *Macrolabis*, *Hartigiola*, *Mikiola*, and *Phegomyia*). Sequence data for the *COI*, 16S rRNA (16S), 28S rRNA (28S), internal transcribed spacer 1 (ITS1), and *carbamoyl‐phosphate synthetase 2*, *aspartate transcarbamylase*, *and dihydroorotase* (*CAD*) of Cecidomyiinae species used in Dorchin et al. (2019) were retrieved from GenBank. These loci include both mitochondrial and nuclear markers, encompassing conserved and variable regions suited for inference at different taxonomic levels in Cecidomyiinae (Dorchin et al. 2019). Sequences for cecidomyiids known to induce galls on *Fagus* were also included, if available: *Contarinia fagi* Rübsaamen (*COI*, JQ684875.1; host, *F. sylvatica* ), *Hartigiola faggalli* (Monzen) (host, *F. crenata* ) of adaxial side‐type isolate M01hf (*COI*, AB753796.1), and abaxial side‐type isolate M23hf (*COI*, AB753817.1). Additionally, four taxa representing the basal subfamilies Catotrichinae ( *Catotricha subobsoleta* ), Micromyinae (*Catocha angulata*), and Porricondylinae (*Asynapta* sp.; *Porricondyla nigripennis* ) were employed as outgroups, as per Dorchin et al. (2019).

Sequences were aligned using MAFFT ver. 7 (Kuraku et al. 2013). *COI* sequences were aligned using the FFT‐NS‐1 progressive algorithm (Katoh et al. 2002), and sequences of other loci were aligned independently using the E‐INS‐i algorithm (Katoh et al. 2005). Poorly aligned regions of *CAD* were trimmed using trimAl ver. 1.50 (Capella‐Gutiérrez et al. 2009), with gap and similarity thresholds of 0.5 and 0.001, respectively. The final lengths of aligned sequences, including gaps, were as follows: 16S, 670 bp (108 taxa); ITS1, 1606 bp (120 taxa); 28S, 754 bp (115 taxa); *COI*, 711 bp (203 taxa); and *CAD*, 752 bp (132 taxa). All loci were then concatenated into a single dataset using MEGA ver. 11.0.11 (Tamura et al. 2021), resulting in a complete dataset containing 216 taxa with a maximum length of 4491 bp. The dataset was then partitioned by locus and further divided according to first, second, and third codon positions for protein‐coding regions.

Phylogenetic analyses were conducted using the maximum‐likelihood (ML) algorithm in IQ‐TREE2 ver. 2.3.4 (Minh et al. 2020). The following substitution models were determined using Modelfinder (Kalyaanamoorthy et al. 2017): 16S, GTR + F + I + G4; ITS1, GTR + F + I + G4; 28S, TVM + F + I + G4; *COI*_1st, GTR + F + I + R5; *CAD*_1st, GTR + F + I + G4; *CAD*_2nd + *COI*_2nd, GTR + F + R3; *COI*_3rd, TPM2u + F + I + R5; and *CAD*_3rd, TIM3 + F + I + R4. Nodal support was evaluated using the Shimodaira–Hasegawa‐like approximate likelihood ratio test (SH‐aLRT) with 1000 replicates (Guindon et al. 2010) and ultrafast bootstrap (UFBoot) analysis with 1000 replicates (Hoang et al. 2018). Default settings were used for other parameters. Finally, the phylogenetic tree was visualized using iTOL ver. 6.8.1 (Letunic and Bork 2024).

### 2.4 Ancestral State Reconstruction

The *Fagus*‐feeding clade was pruned from the ML IQ‐TREE and subjected to ancestral state reconstruction using Mesquite 3.81 (Maddison and Maddison 2023). The Markov k‐state 1 (Mk1) parameter model (Lewis 2001) was used for ML reconstructions, assuming equal probability for any character change. Host plant species ( *F. crenata* , *F. japonica* , *F. orientalis* , or *F. sylvatica* ), gall shape (globoid, horn, discoid, bivalve, or pocket), galling location (lamina, edge, vein, or midrib), and galling side of the leaf (adaxial or abaxial) were coded and reconstructed. One taxon per gall type was included in the analysis.

### 2.5 Divergence Time Estimation

Divergence time was estimated using Bayesian Evolutionary Analysis Sampling Trees (BEAST2) ver. 2.7.7 (Bouckaert et al. 2019). The concatenated dataset was partitioned by locus and codon position, as described above. The bModelTest (Bouckaert and Drummond 2017) with transition‐transversion split was used to identify the most appropriate substitution model for each partition. A Birth‐Death model (Gernhard 2008) was used as the tree prior, with estimation of the relative death rate (*μ*/*λ*) and birth difference rate (*λ* − *μ*). An optimized relaxed clock model (Douglas et al. 2021) under a log‐normal prior distribution was used with an exponentially distributed mean (ORCucldMean.c = 10) and gamma‐distributed standard deviation. Six priors exhibiting log‐normal distribution (M = 1.0, S = 1.25) were applied to the estimated time to the most recent common ancestor (TMRCA), referring to the Paleobiology Database ver. 1.3 (McClennen et al. 2024): genera *Contarinia*, *Lestodiplosis*, and *Clinodiplosis* (amber fossils collected in Mexico, 16.0 Ma; Gagné 1973), tribe Dasineurini, based on *Jaapiella acinaciformis* (amber fossil collected in Ukraine, 33.9 Ma; Fedotova and Perkovsky 2005), subfamily Micromyinae, based on *Eltxo cretaceus* (amber fossil collected in Northern Spain, 99.6 Ma; Arillo and Nel 2000), and subfamily Catotrichinae, based on *Mesotrichoca mesozoica* (sediment fossil collected in Russia, 145.0 Ma; Kovalev 1990; Jaschhof and Jaschhof 2008). Bayesian Markov chain Monte Carlo (MCMC) simulations were repeated for 200 million generations, with sampling every 1000 generations. Log files were analyzed in Tracer ver. 1.7.2 (Rambaut et al. 2018) to examine convergence with reference to the effective sample size (ESS) of each inferred parameter. An ESS > 200 was ensured for all parameters. The first 10% of the generations were discarded as “burn‐in” leaving a total of 180,002 trees remaining. A maximum clade credibility (MCC) tree with 95% highest posterior density (95% HPD) intervals was compiled using TreeAnnotator ver. 2.7.7. The Bayesian inference (BI) time‐calibrated tree was visualized using iTOL ver. 6.8.1 (Letunic and Bork 2024).

### 2.6 Rate Through Time Analysis

Using the R package ‘ape’ ver. 5.8 (Paradis et al. 2004), the *Fagus*‐feeding clade was pruned from the time‐calibrated BI tree to include single representatives of each taxon for each type, resulting in a tree with 28 taxa. This subtree was subjected to lineage‐through‐time (LTT) analysis (Nee et al. 1992) using the R package ‘phytools’ ver. 2.3.0 (Revell 2024; R Core Team 2024). The *γ*‐test was used to infer historical changes in the diversification rate of a clade (Pybus and Harvey 2000), in which *γ* < 0 indicated an increase in diversification rate over time; *γ* = 0 indicated a constant rate of diversification; and *γ* > 0 indicated a decrease in the rate of diversification over time.

### 2.7 Cophylogenetic Analysis

To test for congruence between the phylogenies of *Fagus* host species and their associated gall midges, we conducted a Parafit analysis using the ‘phytools’ package in R. As in the rate‐through‐time analysis, we pruned the time‐calibrated BI tree to retain only the *Fagus*‐feeding clade (28 taxa). For the host phylogeny, we used the *Fagus* species tree from Renner et al. (2016). A global Parafit test was first performed to evaluate the overall host–parasite phylogenetic congruence. ParafitLink1 and ParafitLink2 tests were then performed to assess the significance of individual host–parasite associations.

## 3 Results

### 3.1 Collection of Galls From *Fagus* Forests

A total of 18 types of galls were observed on the leaves of *F. crenata* (8 on Mt. Amagi, 7 on Mt. Gozen, 7 on Mt. Kanyudo, 5 on Mt. Mito, and 11 on Mt. Nabewari), and 11 types were found on the leaves of *F. japonica* (9 on Mt. Gozen and 3 on Mt. Mito), as summarized in Figure 2 and Table 1. Among these, galls **Q** and **R** on *F. crenata* and **Z**–**AC** on *F. japonica* exhibited distinctive morphologies compared to the known types (Yukawa 1991); thereby, they were distinguished from the latter. Among the 29 gall types, 15 types were observed at two or more sites, whereas 14 types were found at only a single site (Table 1). Some types, such as **G** and **O**, were relatively widespread, occurring across multiple mountains, while others (e.g., **A** and **Q**) were restricted to a single locality.

Galls of type **Q** were horn‐shaped with a rounded tip and measured approx. 1 mm in diameter and 3 mm in height (Figure 2Q). The morphology resembled that of **P** (Figure 2P) but differed in terms of galling location; **Q** formed on the edge of the abaxial side of the leaf (Figure 2Q), whereas **P** formed exclusively on the adaxial side of the lamina.

Galls of type **R** were nodular structures that formed at the inward‐curling edge on the abaxial side of leaves and measured 1.5–2 mm in diameter (Figure 2R). Unlike **C** (Figure 2C), which also formed at the leaf margin, **R** exhibited flattened swelling rather than a spherical shape.

Galls of type **Z** were fang‐shaped structures found on the lateral veins (Figure 2Z). These galls developed on the adaxial side of leaves and measured 1–1.5 mm in diameter and 5–6 mm in height, with a smooth surface, pointed tip, and brown color. The morphology was similar to that of **I** on *F. crenata* leaves (Figure 2I).

Galls of type **AA** formed along the lateral veins of leaves, with narrow gaps on the adaxial side (Figure 2AA). Although the swollen morphology resembled that of **A** (Figure 2A) and **T** (Figure 2T), which formed on a section of the lateral vein (6–15 mm long; Yukawa 1991), **AA** extended from the midrib to near the leaf margin.

Galls of type **AB** were onion‐shaped structures on the edge of the abaxial side of the leaf and exhibited a smooth green surface (Figure 2AB). **AB** were similar to the globular **S** on the leaf edge (Figure 2S) but differed by having a pointed tip. **AB** measured approx. 5 mm in diameter and 4 mm in height (Figure 2AB), slightly smaller than **S**.

Galls of type **AC** exhibited a hairy surface and formed on the adaxial side of the leaf (Figure 2AC). These galls were characterized by standing upright above the concave leaf and appeared to be pointed when viewed from the abaxial side. The galls measured 1.2–1.5 mm in diameter and 1.5–2 mm in height. The surface was reddish‐brown, akin to **U**, which were globular with red hair, although the hairs of **AC** were shorter than those of **U** (Figure 2U).

### 3.2 Molecular Phylogeny and Divergence Time

A total of 67 *COI* sequences (654–687 bp) were obtained from cecidomyiids associated with 28 types of galls (**a**–**ac**, excluding **f** due to insufficient PCR amplification). The sequences of most cecidomyiids within the same gall type (**b**, **c**–**e**, **g**–**k**, **o**–**s**, **u**, **w**, **y**, and **aa**–**ac**; *n* = 2–7) exhibited high similarity (> 99.0%), regardless of collection site. Nevertheless, lower similarity was observed for **m** (97.5%; *n* = 2), **n** (93.0%; *n* = 4), and **z** (97.5%; *n* = 2).

The *COI* sequence from **e** and **h**, which induce bivalve‐shaped galls, showed high homology to *H. faggalli*, the reported inducer of **E** (98.6% identity to isolate M01hf; GenBank accession, AB753796.1) and **H** (98.4% identity to isolate M23hf; GenBank accession, AB753817.1), respectively. By contrast, the sequence from **v** exhibited only 90.0% identity with that of *Hartigiola annulipes* (GenBank accession, MN191300.1), with a sequence difference of 60 of 607 bp. Although the inducer of **V** is believed to be *Hartigiola annulipes* (Yukawa et al. 2021), the species also reportedly induces galls on *F. sylvatica* (Pilichowski and Giertych 2020); this apparent discrepancy raises questions regarding the taxonomic assignment of the **V** inducer.

**FIGURE 3:**
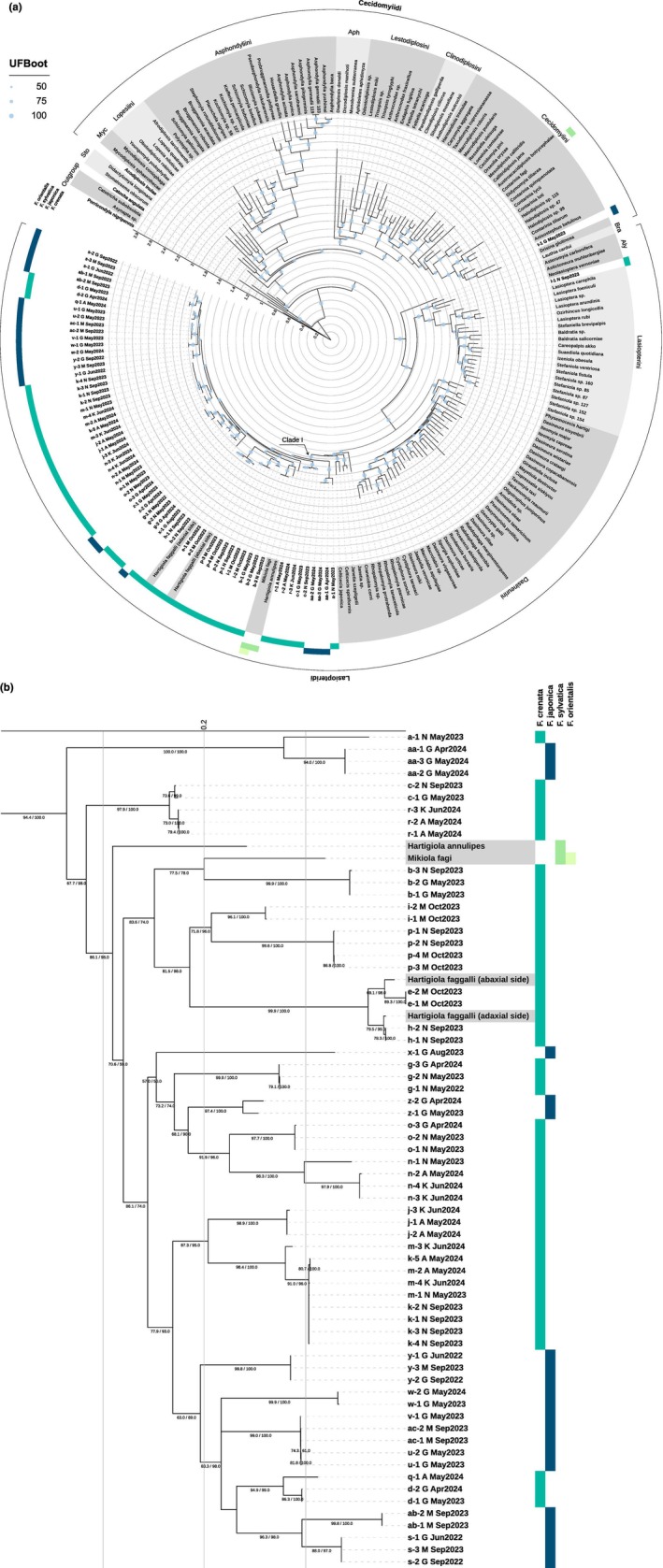
ML phylogenetic tree of cecidomyiids galling on *F. crenata* and *F. japonica* and known taxa of Cecidomyiinae based on concatenated *COI*, 16S, ITS1, 28S, and *CAD* sequences. (a) Whole tree and (b) pruned subtree (Clade I). Hyphenated values indicate individual specimens collected from different galls. The capital letters A, G, K, and M following taxa **a**–**ac** indicate the collection sites: A, Mt. Amagi; G, Mt. Gozen; K, Kanyudo; M, Mt. Mito; N, Mt. Nabewari. Nodal support indicated by UFBoot values (1000 replications) and SH‐aLRT values (1000 replications). Tribes: Bra, Brachineuridi; Myc, Mycodiplosini; Sto, Stomatosematidi. Strips around the tree indicate the host *Fagus* species, in the order *F. crenata* , *F. japonica* , *F. sylvatica* , and *F. orientalis* from the inside.

**FIGURE 4:**
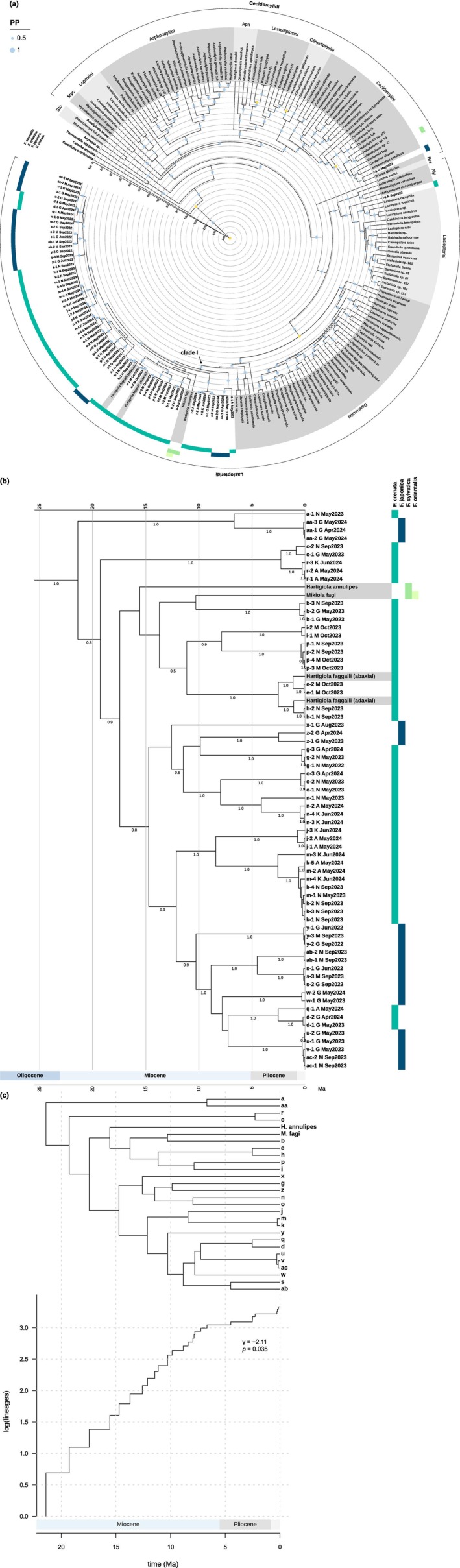
BI time‐calibrated phylogenetic tree of cecidomyiids galling on *F. crenata* and *F. japonica* and known taxa of Cecidomyiinae based on concatenated *COI*, 16S, ITS1, 28S, and *CAD* sequences. Note that the sequences of gall midges labeled **a–ac** are based solely on *COI* data. Support for branches is indicated by PP values above branches. Yellow circles indicate nodes used as calibrated priors in divergence time estimation in BEAST2. Nodes are at mean divergence times. The time scale is Ma. (a) Whole tree and (b) pruned subtree (Clade I). Hyphenated values indicate individual specimens collected from different galls. The capital letters A, G, K, and M following taxa **a**–**ac** indicate the collection sites: A, Mt. Amagi; G, Mt. Gozen; K, Kanyudo; M, Mt. Mito; N, Mt. Nabewari. (c) LTT plot of the phylogeny of the *Fagus*‐feeding lineage (Clade I). The natural logarithm of the number of lineages versus time since the origin of the lineage (Ma) is shown with the γ statistic and corresponding *p* value.

Phylogenetic analyses of 67 OTUs and 149 known species produced congruent topologies in both the ML and BI trees (Figures 3 and 4). The supertribe Lasiopteridi was clearly divided into three tribes: Dasineurini, Lasiopterini, and Alycaulini, which were fully supported (SH‐aLRT = 99.8 and UFBoot = 100 in ML; PP = 1 in BI). With the exception of *F. japonica* ‐feeding **t**, which was classified into tribe Cecidomyiini in Cecidomyiidi, all leaf gallers feeding on *F. crenata* and *F. japonica* trees were assigned to the Lasiopteridi. Within the Lasiopteridi clade, all Japanese *Fagus*‐feeding taxa other than **l** were assigned to Dasineurini, alongside previously described *Fagus*‐feeding taxa (*Hartigiola annulipes*, *Hartigiola faggalli*, *Mikiola fagi*, and *Mikiola bicornis*). Only cecidomyiid **l** occupied a basal position relative to tribe Lasiopterini, which was assumed to be ‘ambrosia gallers’ associated with symbiotic fungi (Gagné and Jaschhof 2021).

Within the *Fagus*‐feeding clade of tribe Dasineurini (Figures 3b and 4b), most nodes were well supported (SH‐aLRT ≥ 80%, UFBoot ≥ 95%, PP ≥ 0.9). The phylogenetic positions of *Hartigiola faggalli* and *Hartigiola annulipes* indicate that the genus *Hartigiola* is paraphyletic. Although support for the monophyly of *Mikiola fagi* and **b** (described as *Mikiola bicornis*) was relatively low (SH‐aLRT = 77.5, UFBoot = 78, PP = 0.4), these appeared to be sister species. Some relationships among taxa remained unresolved. The monophyly of *F. crenata* ‐feeding **k** and **m** was highly supported (SH‐aLRT = 98.4, UFBoot = 100, PP = 1), suggesting a possible sister relationship; however, they were intermixed within a single clade. The relationships among *F. japonica* ‐feeding **u**, **v**, and **ac** also could not be clearly resolved.

Within the tribe Dasineurini (Figure 4a), the *Fagus*‐feeding lineage and *Janetia*, gall inducers feeding on *Quercus* (Fagaceae; Gagné and Jaschhof 2021) were estimated to have diverged in the late Oligocene period (ca. 25.4 Ma; 95% HPD, 30.14–20.71 Ma). The crown age of the *Fagus*‐hosing taxa was inferred at the beginning of the Miocene period (Figure 4b; ca. 21.4 Ma; 95% HPD, 26.07–16.77 Ma). Along with the divergences of West Eurasian *Fagus*‐feeding species, *Hartigiola annulipes* (ca. 15.6 Ma; 95% HPD, 19.42–11.73 Ma) and *Mikiola fagi* (ca. 10.3 Ma; 95% HPD, 14.95–5.86 Ma), the Japanese *Fagus*‐feeding taxa rapidly radiated during the Miocene and beyond. Ongoing diversification is further supported by the LTT plot and the negative γ statistic (Figure 4c; *γ* = −2.11, *p* = 0.035).

### 3.3 Evolution of Gall Characteristics

**FIGURE 5:**
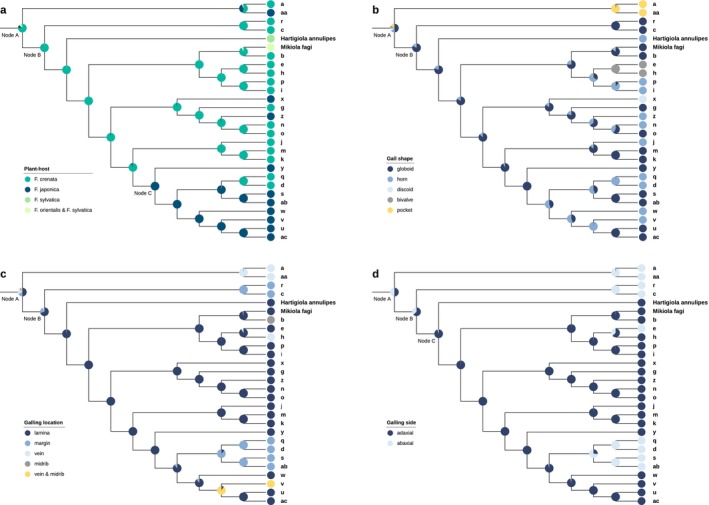
Ancestral state reconstructions for *Fagus*‐feeding taxa in Dasineurini using ML and stored Mk1 models. Areas of pie charts indicate relative support for ancestral states. (a) Host plant species. (b) Gall shape. (c) Galling location on the leaf. (d) Galling side of the leaf.

Likelihood‐based ancestral character states for host plant species, gall shape, galling location, and leaf side (adaxial or abaxial) are presented in Figure 5. Reconstruction of the evolutionary history of host plant use indicated that the root‐ancestral host was of the *F. crenata* lineage (76.6%; Figure 5a). Other potential ancestral hosts were identified with significantly lower probabilities (10.6% for *F. japonica* and approx. 6% for other *Fagus* species). Among 28 taxa studied, *F. crenata* remained the host for 16 taxa. The phylogenetic positions of the *F. japonica* ‐feeding taxa **aa**, **x** and **z**, and **s**, **u**–**w**, **y**, **ab**, and **ac** suggest that at least three independent host shifts from *F. crenata* to *F. japonica* occurred. Additionally, the positions of **d** and **q** suggest that their common ancestor shifted back to *F. crenata* . Host shifts to the West Eurasian beech species *F. sylvatica* and *F. orientalis* may have also occurred once or twice.

The reconstruction of gall shapes suggested that the globular shape is the most probable ancestral state (Figure 5b), although it was not highly favored at Node A (globoid, 48.1%, with other shapes ranging from 10% to 15%). The vein pocket shape (**a**, **aa**), which occupies a basal position, likely evolved once from an uncertain ancestral state. At Node B, globoid was more strongly favored (82.0%), followed by independent evolution of the horn shape five times and single occurrences of the bivalve (**e**, **h**; *Hartigiola faggalli*) and discoid (**x**) shapes.

Regarding the evolution of galling location (Figure 5c), Node A showed nearly 50% probability for lamina (49.5%), 20.0% for vein, 18.9% for leaf margin, and < 6% for others. Although the ancestral location at the basal node was not clearly attributed, lamina was the most probable (70.7%) at Node B, with leaf margin (22.2%) and others (≥ 4%) being less likely. Of the 26 descendant taxa, the lamina is still the galling site of 17. Location shifts from the lamina to leaf margin likely occurred once or twice. The basal taxa **a** and **aa** exclusively form galls at the side vein, and midrib and/or side vein galling evolved independently several times in more recent groups.

At the root node, the probability of adaxial versus abaxial galling was approximately equal (50%); however, several basal nodes exhibited a higher probability of adaxial galling (Figure 5d). The probability of galling on the adaxial side was 63.2% at Node B and 95.6% at Node C, suggesting that adaxial gall formation was the ancestral state in Clades B and C. Multiple independent shifts to the abaxial side occurred across the phylogeny, often accompanied by a shift to the leaf margin.

### 3.4 Cophylogenetic Signal Between Gall Midges and Host Plants

**FIGURE 6:**
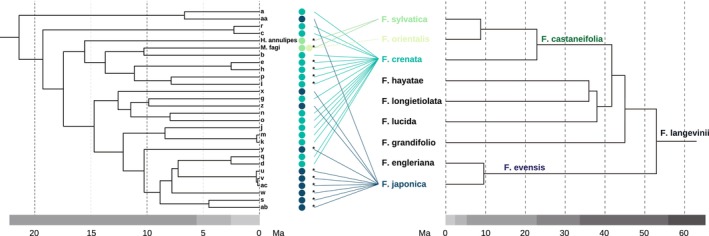
Tanglegram comparing the evolutionary histories of *Fagus*‐feeding cecidomyiids in the tribe Dasineurini and *Fagus* host species. The host phylogeny is adapted from Renner et al. (2016). ParafitGlobal, *p* = 0.011. *p < 0.05 in Parafit Link1 and Link2 tests.

Parafit analysis detected a significant global phylogenetic congruence between *Fagus*‐feeding gall midges and their host *Fagus* species (ParafitGlobal, *p* = 0.011; Figure 6). Among the 28 host–parasite links, 13 links contributed significantly to the overall cophylogenetic structure in Parafit Link1 and Link2 tests (*p* < 0.05), involving all *Fagus* species that host cecidomyiids (Figure 6). The *p* values of Link1 and Link2 tests were nearly identical in all cases.

## 4 Discussion

### 4.1 Phylogenetic Positions of *Fagus*‐Feeding Cecidomyiids

The ML and BI phylogenetic analyses illustrated the presence of robust divisions within the subfamily Cecidomyiinae and provided insights into the phylogenetic relationships between cecidomyiids associated with *Fagus* trees. The taxonomic assignment of leaf gallers collected from both *F. crenata* and *F. japonica* into tribes Dasineurini and Lasiopterini was consistent with Yukawa (1991), who speculated that the majority of cecidomyiids associated with Japanese *Fagus* trees likely belong to the supertribe Lasiopteridi.

Cecidomyiids **a**–**e**, **g**–**k**, **m**–**s**, and **u**–**ac** belong to the same lineage, along with the described species of *Hartigiola* and *Mikiola* inhabiting *F. orientalis* and *F. sylvatica* . Although the generic level cannot be determined solely based on molecular phylogeny, leaf gallers **a**–**e**, **g**–**k**, **m**–**s**, and **u**–**ac** most likely belong to or are closely related to the genera *Hartigiola* or *Mikiola*. These putative congeners may have radiated on *Fagus* leaves, as discussed in the following section. The inducer **v** is also likely to belong to *Hartigiola* or *Mikiola*, but appears to differ from *H. annulipes* , despite previous reports (Yukawa et al. 2021), due to substantial sequence divergence in the *COI* and its association with a different host species ( *F. japonica* vs. *F. sylvatica* ). To clarify the taxonomic identity of **v** at the species level, morphological comparisons and molecular analyses using multiple independent loci between **v** and *H. annulipes* will be necessary to evaluate their taxonomic distinctiveness. Alternatively, the GenBank sequence of *H. annulipes* may represent a misidentified specimen.

The pocket vein gall‐inducer **t**, classified within tribe Cecidomyiini, may be related to *Contarinia fagi*, a bud galler inhabiting *F. sylvatica* , in addition to several other *Contarinia* species. *Contarinia* is a highly diverse genus within Cecidomyiini and currently includes over 300 described species worldwide and serves as a catch‐all genus for the tribe (Gagné and Jaschhof 2021). Although *Contarinia* species are associated with a wide range of plant families, including flower buds and leaves (Yukawa et al. 2005), they have not been previously reported on *F. crenata* or *F. japonica* .

Although we included reference sequences from multiple genes retrieved from public databases, our newly generated data were restricted to mitochondrial *COI*, which provides limited resolution among recently diverged taxa and is susceptible to introgression. Future studies incorporating both broader within‐type sampling and additional nuclear markers will be essential for evaluating cryptic diversity and refining phylogeny.

### 4.2 Gall Morphology in Relation to the Phylogenetic Positions of Inducers

Gall morphology is often regarded as an extended phenotype of galling insects (reviewed in Stone and Schönrogge 2003). Previous studies on aphids (Stern 1995), thrips (Crespi and Worobey 1998), wasps (Stone and Cook 1998), and sawflies (Nyman et al. 2000) suggested that galling insects exert control over the key aspects of gall morphology. The diverse array of gall morphologies observed on the single host plants *F. crenata* and *F. japonica* can be attributed to factors originating from the cecidomyiids, rather than to constraints imposed by the host plant.

Although galls induced by closely related taxa do not always display similar morphologies (e.g., **N** and **O**), sister taxa often exhibit similar gall morphologies in analogous foliar locations (i.e., **C** and **R** on *F. crenata* ; **I** and **P** on *F. crenata* ; **D** and **Q** on *F. crenata* ; **S** and **AB** on *F. japonica* ; **U**, **V**, and **AC** on *F. japonica* ). In some cases, this pattern persists even after a host shift occurs, as seen with the similarity between gall morphologies on *F. crenata* ‐**A** and *F. japonica* ‐**AA** and the galls induced by *Mikiola fagi* on *F. orientalis* and *F. sylvatica* and *Mi. bicornis*‐induced **B** on *F. crenata* (Ellis 2001). These similar or analogous morphologies may arise from shared behavioral characteristics, such as oviposition and/or larval feeding activity (Ferreira et al. 2019). As inferred from ancestral state reconstruction analyses, the horn‐shaped morphology has evolved repeatedly from ancestral taxa that produce globular galls. Convergent evolution may involve adaptive significance such as enhancement of nutritive supply and/or defense against predators and pathogens (Joy and Crespi 2007; Stireman III et al. 2012).

In the ancestral state reconstruction, we used a single representative per gall type to simplify trait assignment and ensure comparability across taxa. While *COI* sequences were nearly identical among individuals of the same type in most cases, several types (e.g., **M**, **N**, and **Z**) exhibited within‐type variation. This approach may therefore overlook cryptic genetic diversity among inducers that form morphologically similar galls, potentially underestimating trait transitions or polymorphisms. Future studies aiming to address such hidden diversity may benefit from broader within‐type sampling and the incorporation of additional genetic markers.

### 4.3 Feeding Modes of *Fagus*‐Feeding Cecidomyiids

Larval feeding modes in the subfamily Cecidomyiinae are considered to have evolved from ancestral fungivory to herbivory, ambrosia, and predation. The basal supertribes Stomatosematidi and Brachyneuridi, along with the tribe Mycodiplosini within Cecidomyiidi, retain the fungivorous habit (Dorchin et al. 2019). Although some taxa in the Clinodiplosini have reverted from herbivory to fungivory, plant‐feeders represent the most species‐rich group, having undergone explosive diversification between 25 and 50 Ma (Dorchin et al. 2019). Tribes Dasineurini and Cecidomyiini are exclusively herbivorous (Gagné and Jaschhof 2021), suggesting that **a**–**ac** (except for **f**, **l**, and **t**) are herbivorous. Notably, no cecidomyiids with *COI* sequences identical to those of inducers found on *F. crenata* were found on *F. japonica* , even in areas in which these *Fagus* trees exist sympatrically. This discrepancy suggests that cecidomyiid species found on Mt. Gozen and Mt. Mito are monophagous and associate exclusively with either *F. crenata* or *F. japonica* .

### 4.4 Biogeographical and Evolutionary History of *Fagus* and Associated Gall Inducers

Cophylogenetic analysis revealed a significant overall congruence between the phylogenies of *Fagus* species and their associated gall midges (Figure 6). Multiple host–parasite associations underpinned this pattern of phylogenetic congruence. This suggests that a combination of co‐divergence, host shifts, and within‐host radiations has shaped the evolutionary diversification of *Fagus*‐associated cecidomyiids. These results align with the biogeographical and evolutionary history of *Fagus*, which underwent major diversification events during the Miocene and Pliocene in both East Asia and Europe. The divergence of the West Eurasian *Fagus*‐feeding species *Hartigiola annulipes* (ca. 15.6 Ma) and *Mikiola fagi* (ca. 10.3 Ma) from the *F. crenata* ‐feeding lineage suggests that their ancestral host trees coexisted in the same geographical region during the middle Miocene period (Figures 4 and 5a). Such host transitions are likely to have reflected and been facilitated by the biogeographic dynamics of *Fagus* species, as indicated by the following palaeobotanical and molecular phylogenetic studies.

**FIGURE 7:**
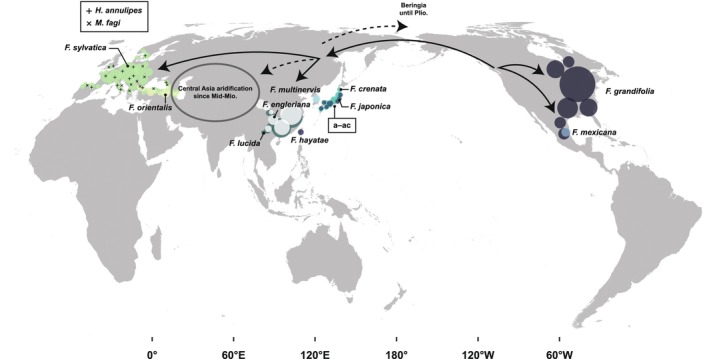
Hypothetical migratory routes of *Fagus* and associated gall inducers. Species distributions of major accepted *Fagus* species (Tanaka and Matsui 2007; Rodríguez‐Ramírez et al. 2013; EUFORGEN 2024; POWO 2025) are shown by circles. Symbols + and × indicate the distribution of *Fagus*‐feeding gall midges currently reported in Europe (i.e., *Hartigiola annulipes* and *Mikiola fagi*, respectively; GBIF Secretariat, 2023). **a**–**ac**, Japanese *Fagus*‐feeding gall midges (see Table 1). Solid arrows indicate the migratory direction of ancestral *Fagus* species. Dashed arrows indicate routes in which migration likely did not occur; or, if migration occurred, it was likely associated with a range reduction due to factors such as the aridification of Central Asia. Mid‐Mio., middle Miocene period; Plio., Pliocene period.

The genus *Fagus* originated in the North Pacific region during the early Eocene period (ca. 50 Ma; Manchester and Dillhoff 2004), and by the Pliocene period (5.3–2.6 Ma), *Fagus* had disappeared from western North America (Smiley 1963), whereas extant species, *F. grandifolia* and *F. mexicana* , persisted in the southern and eastern regions without any association with gall midges (Figure 7). By contrast, the derivative species migrated to the Eurasian continent across Beringia (Figure 7; Denk and Grimm 2009). The subgen. *Engleriana* diverged from the subgen. *Fagus* in the North Pacific region ca. 63–43 Ma (Renner et al. 2016). Unlike subgen. *Fagus*, the range of subgen. *Engleriana* seems to have remained confined to Northeast Asia (Uemura 2002). After closure of the Turgai Strait ca. 37 Ma, the range of *Fagus* expanded westward during the early Oligocene period (Denk and Grimm 2009). By the early/middle Miocene, a single species, *F. castaneifolia* Unger, was prevalent throughout the Northern Hemisphere, forming a homogenous biota (Denk 2004). Coinciding with the expansion of the range of *F. castaneifolia*, the common ancestor of the *Fagus*‐feeding lineage in tribe Dasineurini diverged from other Fagaceae‐feeding taxa (e.g., *Janetia*) approximately 30.1–20.7 Ma (Figure 4), presumably originating in Eurasia. The most ancestral host of the *Fagus*‐feeding lineage in tribe Dasineurini might be *F. castaneifolia*.

After the middle Miocene period, global cooling and uplift of the Himalaya‐Tibetan Plateau led to aridification in Central Asia (An et al. 2001; Miao et al. 2012; Ding et al. 2022). This climatic shift likely disrupted the formerly homogeneous distribution of *Fagus*, driving its retreat into East Asia and West Eurasia (Jiang et al. 2022). Such geographic fragmentation may have triggered the divergence of subgen. *Fagus* into the Northeast Asian, Central European, and Turkish lineages. Indeed, previous molecular phylogenetic studies inferred a sister relationship between *F. crenata* and the West European lineages ( *F. orientalis* + *F. sylvatica* ; Denk et al. 2005; Renner et al. 2016; Cardoni et al. 2022). The ecological segregation of *Fagus* hosts would have isolated their associated cecidomyiids, promoting regional co‐diversification. Notably, the earliest fossil record of a *Fagus* gall, found in Miocene strata in Spain, was attributed to the extinct *Mikiola pontiensis* Villalta on *F. castaneifolia* (Diéguez et al. 1996). The LTT plot suggests that the *Fagus*‐feeding lineage in the tribe Dasineurini underwent adaptive radiation during the middle Miocene, a period characterized by major climatic and tectonic shifts in the Northern Hemisphere that may have catalyzed diversification in both hosts and gall inducers (Figure 4c).

During the early Miocene period, Japan was separated from the Eurasian continent and transformed into an archipelago through continental uplift ca. 25–13 Ma (Jolivet et al. 1994). The opening of the Sea of Japan led to ecological segregation between the Eurasian continent and the Japanese Archipelago, where distinct niches fostered the development of a unique assemblage of *Fagus* flora and associated cecidomyiid fauna. In this period, Japanese beech flora included several species, such as *F. stuxbergi* (Nathorst) Tanai (subgen. *Fagus*), *F. palaeocrenata* Okutsu (subgen. *Fagus*), and *F. palaeojaponica* Suzuki (subgen. *Engleriana*), which likely led to the modern *F. hayatae*, *F. crenata* , and *F. japonica* , respectively (Okutsu 1955; Tanai 1974). Although details regarding the evolutionary processes that led to modern *Fagus* species remain elusive (Momohara and Ito 2023; Hara 2024), Japanese *Fagus*‐feeding leaf gallers likely coevolved with these relict *Fagus* hosts in the eastern Palearctic realm, particularly in the segregated Japanese Archipelago during the Neogene period.

### 4.5 Possible Mechanisms of Speciation With/Without Host Shifts

The diversification of *Fagus*‐feeding gall inducers within tribe Dasineurini appears to have been driven by host and location shifts on the leaves, including both the adaxial and abaxial surfaces. The lineage of cecidomyiids associated with *Fagus* seems to have originally inhabited on *F. castaneifolia*. During radiation, these gall inducers likely shifted hosts between the *F. crenata* and *F. japonica* lineages in Japan, where these two lineages coexisted parapatrically during the Miocene period.

Adaptive radiation on the single host species *F. crenata* gave rise to taxa such as **b**, **g**, **i**–**k**, and **m**–**p**. The common ancestor of Node C shown in Figure 5a likely originated through a host shift to a non‐natal congener *F. japonica* and subsequently radiated on this host, giving rise to taxa **s**, **u**–**w**, **y**, **ab**, and **ac**. Some taxa, such as **d** and **q**, appear to have shifted back to *F. crenata* from *F. japonica* . Additionally, several nested taxa, such as **x** and **z**, which are associated with *F. japonica* , are included in a clade predominantly associated with *F. crenata* . The occurrence of these nested taxa may also have been accompanied by host plant shifts in regions in which both species coexist parapatrically or sympatrically.

Host shifts to non‐natal species can result from opportunistic ovipositional mistakes, a phenomenon observed in various taxa of herbivorous insects, including many Cecidomyiidae (Price 2005). Through host exchange experiments, Yukawa et al. (2021) demonstrated that the monophagous gall midge *Daphnephila machilicola*, which natively induces leaf galls on *Machilus thunbergii*, lays its eggs on the leaves of a congener, *Machilus japonica*, resulting in the formation of galls with different morphology. Host plant shifts typically entail adaptations to distinct plant characteristics related to morphology, chemistry, and phenology (Jaenike 1989, 1990; Becerra and Venable 1999; Cook et al. 2002). Given that congeners often share various traits, such as chemical profiles (e.g., leaf volatiles; Boddum et al. 2018; Molnár et al. 2018) and morphological characteristics (e.g., leaf thickness) that potentially affect host‐choice behavior, host shifts are expected to occur readily.

Speciation can occur even in the absence of host plant shifts; within‐host speciation may instead arise from shifts in the feeding niche, such as a shift from leaf to stem (Joy and Crespi 2007). Developmental discrepancies among plant organs involve potential phenological separation (Condon and Steck 1997; Després et al. 2002; Ferdy et al. 2002). However, the *Fagus*‐feeding taxa diverged exclusively on the leaves without any organ shifts. Some taxa may have diverged through fine‐scale location shifts within leaves. For instance, the bivalve‐shaped gall inducers **e** and **h** seem to be in the process of speciation through a shift in galling location between the adaxial and abaxial sides of the leaf (Sato and Yukawa 2004; Mishima et al. 2014). Differences in microenvironmental heterogeneity (e.g., sunlight exposure, humidity, or surface chemistry) across a single leaf may promote divergent selection on oviposition behavior and larval performance. In addition, another possible driver of inducer divergence is adaptation to different age classes of a single host plant species. Zhang et al. (2015) suggested that adaptation to different ages (e.g., seedling versus mature tree) of host elm trees ( *Ulmus pumila* L.) can lead to the divergence of sympatric sister species of the elm leaf beetles *Pyrrhalta maculicollis* and *Pyrrhalta aenescens*, likely due to differences in the chemical profiles of the leaf surface at different tree ages. A gradual shift in the allocation of metabolic energy from the roots in seedlings to the shoots in mature trees was demonstrated in *F. crenata* (Kurosawa et al. 2023). Such ontogenetic changes in host plant leaves could impact the fitness of phytophagous cecidomyiids via age‐specific differences in the nutritional content of the leaves. Similar patterns of ecological divergence within a single host species—often involving microhabitat or phenological shifts—have been documented in herbivorous insects beyond cecidomyiids (Tilmon 2008). For example, Kobayashi et al. (2018) reported two sympatric species of Hawaiian leaf‐mining moths (*Philodoria*, Gracillariidae) that diverged by specializing on different parts of the same host plant, *Myrsine* (Primulaceae). Forister et al. (2012) emphasized that ecological specialization—despite incomplete reproductive isolation—can promote divergence, particularly when coupled with behavioral adaptation or complex biotic interactions. Such processes may foster conditions favorable to sympatric speciation, especially in systems shaped by genotype–environment interactions and spatially structured selective pressures.

## 5 Concluding Remarks

This study provides the first molecular phylogeny of cecidomyiids inhabiting *F. crenata* and *F. japonica* . With few exceptions, most *Fagus*‐feeding species share a common lineage, likely belonging to or closely related to *Hartigiola* or *Mikiola* within the tribe Dasineurini, underscoring their herbivorous nature. These cecidomyiids appear to have undergone adaptive radiation on relict *Fagus* species in Japan since the Miocene epoch (ca. ~21 Ma), coinciding with the separation of the Japanese Archipelago from the Eurasian continent and the development of the Asia monsoonal climate. This radiation was characterized by (1) bidirectional host plant shifts between *F. crenata* and *F. japonica* , and (2) fine‐scale location shifts within the leaves. Future comparative studies of the morphological, anatomical, and phenological features of galls and cecidomyiids should yield further insights into the phylogeny and evolutionary context. Additionally, investigation of galls formed on other *Fagus* species, including *F. orientalis* and *F. sylvatica* , will be necessary in order to elucidate the co‐evolutionary relationships with the genus.

## Author Contributions

**Shinnosuke Mori:** conceptualization (supporting), data curation (lead), formal analysis (lead), investigation (lead), methodology (lead), software (lead), visualization (lead), writing – original draft (lead), writing – review and editing (equal). **Yugo Dhakhwa:** investigation (supporting). **Makoto Tokuda:** methodology (supporting), writing – review and editing (equal). **Yoko Saikawa:** conceptualization (lead), funding acquisition (lead), project administration (lead), writing – original draft (supporting), writing – review and editing (equal).

## Conflicts of Interest

The authors declare no conflicts of interest.

## Supporting information


Table S1.

## Data Availability

Sequence data in this study are available on the NCBI GenBank accession PQ838091–PQ838157.
